# Serotonin syndrome risk with concomitant opioid and serotonergic antidepressant use: a multinational pharmacovigilance study

**DOI:** 10.3389/fphar.2026.1773721

**Published:** 2026-03-18

**Authors:** Junge Zhang, Wenxin Wang, Hehe Wang, Changshun Huang, Lifeng Yao, Rui Zhang, Jialei Chen

**Affiliations:** 1 Department of Anesthesiology, The First Affiliated Hospital of Ningbo University, Ningbo, China; 2 Department of Otolaryngology, Head and Neck Surgery, The First Affiliated Hospital of Ningbo University, Ningbo, China; 3 Ningbo Mingzhou Hospital Co., Ltd., Ningbo, China

**Keywords:** serotonin syndrome, opioids, serotonergic antidepressants, pharmacovigilance, drug interaction, perioperative period

## Abstract

**Background:**

The concurrent use of serotonergic antidepressants (SSRIs, SNRIs) and opioid analgesics is frequent in the perioperative setting. While this combination carries a known risk of serotonin syndrome, large-scale epidemiological evidence remains scarce.

**Methods:**

We performed a multinational pharmacovigilance study using data from the US FDA Adverse Event Reporting System (FAERS) and the Japanese Adverse Drug Event Report database (JADER) from Q1 2004 to Q2 2025. Disproportionality analyses, including Reporting Odds Ratio (ROR) with a multi-algorithm framework, were employed to quantify signals for serotonin syndrome and other adverse events. We further characterized clinical profiles and conducted gender-stratified and time-to-onset analyses.

**Results:**

Opioids (e.g., tramadol, fentanyl, oxycodone) were consistently among the top drugs associated with serotonin syndrome reports. Concomitant SSRI/SNRI-opioid use showed exceptionally strong signals for serotonin syndrome (SSRI-opioid ROR 95.94, 95% CI 88.9–103.53; SNRI-opioid ROR 57.68, 95% CI 52.32–63.59). SSRI-opioid combinations were uniquely associated with cardiac disorders (ROR 1.87, 95% CI 1.77–1.98), whereas SNRI-opioid use was linked to drug withdrawal syndrome. Gender-stratified analysis indicated a higher reporting proportion for serotonin syndrome in males prescribed SSRI-opioids. Notably, up to 30% of adverse events occurred more than 300 days after therapy initiation, indicating a persistent risk.

**Conclusion:**

This study provides robust multinational evidence that concomitant serotonergic antidepressant and opioid use is strongly associated with a high risk of serotonin syndrome and distinct adverse event profiles. These findings highlight the urgent need for systematic preoperative medication review, tailored perioperative monitoring, and sustained clinical vigilance.

## Background

The management of acute postoperative pain remains fundamentally reliant on opioid analgesics, despite growing initiatives to promote multimodal analgesia (Wick et al., 2017). This persistent dependence on opioids intersects with another major public health trend: the rising global prevalence of major depressive and anxiety disorders, which has led to the widespread use of serotonergic antidepressants, particularly selective serotonin reuptake inhibitors (SSRIs) and serotonin-norepinephrine reuptake inhibitors (SNRIs) (Nawaz et al., 2024; Santomauro et al., 2021; Rong et al., 2025). Consequently, the co-administration of these psychotropic agents with opioids in the perioperative period has become commonplace. This therapeutic intersection, however, is pharmacologically precarious. Both drug classes independently modulate the serotonergic system, creating a potential for synergistic overstimulation of central and peripheral serotonin receptors, which may culminate in serotonin syndrome (Li et al., 2020; Zweckstetter et al., 2021; Dionisie et al., 2021).

Serotonin syndrome is a potentially life-threatening condition characterised by a triad of neuromuscular excitation, autonomic hyperactivity, and altered mental status (Scotton et al., 2019; Francescangeli et al., 2019; Maitland and Baker, 2022). While recognised as a rare complication, its true incidence in the perioperative setting is poorly quantified and likely under-diagnosed, as its symptoms can be misattributed to postoperative agitation, infection, or other causes. The syndrome is classically associated with the combination of two or more serotonergic agents (Buckley et al., 2014). Of particular concern for anaesthetists is the fact that several commonly administered opioids—notably tramadol, tapentadol, methadone, fentanyl, and oxycodone—possess secondary mechanisms of serotonin reuptake inhibition or release, thereby classifying them as potential contributors to serotonergic toxicity (Baldo and Rose, 2020; Baldo, 2021).

Existing evidence on this interaction predominantly stems from case reports and small case series, leaving critical questions unanswered. The magnitude of the risk signal in large, representative populations, the potential differences in risk profiles between SSRI and SNRI subclasses, and the specific clinical manifestations beyond the core symptoms of serotonin syndrome remain poorly delineated. This knowledge gap impedes the development of evidence-based preoperative risk stratification and tailored intraoperative management. Robust, data-driven guidance is urgently needed to inform anaesthetic drug selection and postoperative monitoring protocols for the substantial number of surgical patients on chronic serotonergic therapy.

Therefore, we conducted a multinational pharmacovigilance study utilising extensive data from the US Food and Drug Administration Adverse Event Reporting System (FAERS) and the Japanese Adverse Drug Event Report database (JADER) (Sakaeda et al., 2013; Janiczak et al., 2025). Our primary aims were to: (1) quantify the strength of the association between concomitant serotonergic antidepressant and opioid use and the reporting of serotonin syndrome; (2) characterise and contrast the comprehensive adverse event profiles, including organ system involvement, for SSRI-opioid and SNRI-opioid combinations; and (3) analyse temporal patterns and potential gender-based differences in reporting to provide a nuanced risk assessment specifically tailored to the perioperative context.

## Methods

### Study design and data sources

**FIGURE 1:**
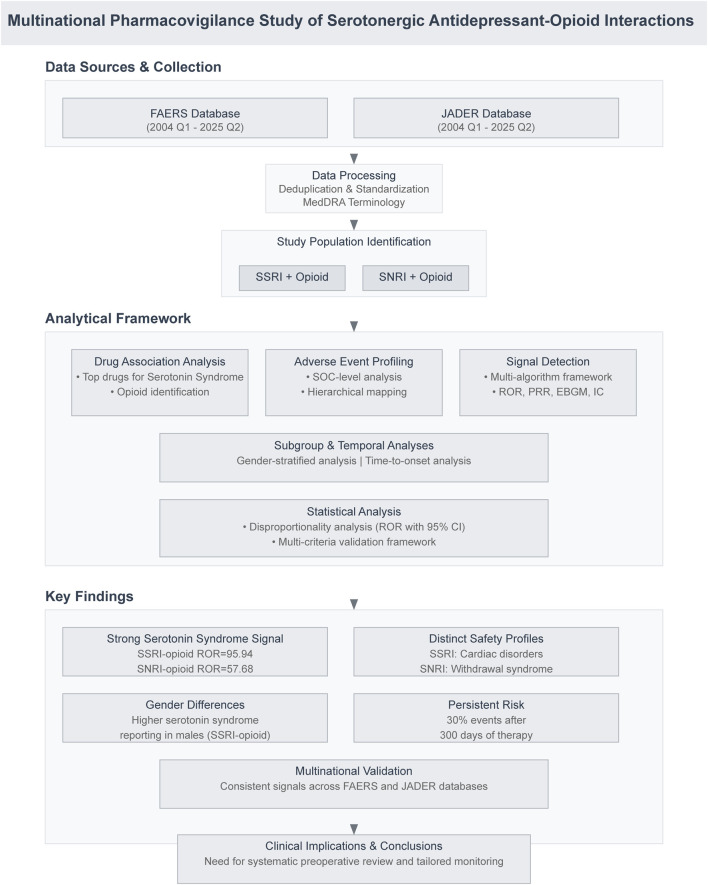
Flowchart of the multinational pharmacovigilance study. Data from FAERS and JADER (2004–2025) were processed and analyzed to identify concomitant SSRI/SNRI-opioid users. Analyses included drug associations, adverse event profiling, signal detection, subgroup and temporal analyses, and statistical validation. Key findings revealed strong serotonin syndrome signals, distinct safety profiles, gender differences, and persistent risk, validated across databases.

This multinational pharmacovigilance study utilized spontaneous adverse event reports from FAERS and JADER, covering the period from the first quarter of 2004 through the second quarter of 2025. These two databases were specifically selected to provide a comparative perspective between Western (FAERS) and Asian (JADER) populations, reflecting distinct genetic backgrounds and prescribing practices regarding opioid use. While our analysis encompasses all reported indications across the entire dataset without restricting to specific perioperative codes, the findings are particularly relevant to the perioperative setting where the co-administration of these analgesic and psychotropic agents is most prevalent. The overall study design is presented in Figure 1.

### Data processing and case definition

To characterize the landscape of medications associated with serotonin syndrome, we identified all reports where serotonin syndrome was coded as an adverse event using the Medical Dictionary for Regulatory Activities (MedDRA) preferred term “serotonin syndrome.” (Brown et al., 1999) For the interaction analysis, we identified cases involving the concurrent use of serotonergic antidepressants (SSRIs or SNRIs) and opioid analgesics. To prioritize the specificity of the detected signals and reduce false positives, we restricted our inclusion criteria to cases where both drug classes were explicitly listed as “Primary Suspect” or “Interacting” by the reporter. While this conservative approach may underestimate the total volume of co-exposure by excluding drugs listed merely as “Concomitant,” it ensures a higher likelihood that the reported adverse event was clinically attributed to the drug combination in question. Regarding missing data, reports lacking critical drug identifiers were excluded; however, reports with missing demographic variables (e.g., age, sex, weight) were retained and categorized as “Unknown” or “Missing” to preserve the maximum sample size for statistical signal detection.

### Assessment of concomitant serotonergic antidepressant and opioid use

Given the frequent reporting of opioids in serotonin syndrome cases, we specifically examined reports involving concurrent use of serotonergic antidepressants (SSRIs or SNRIs) and opioid analgesics. Cases were included only if both drug classes were listed as “primary suspect” or “interacting.” We extracted demographic variables (age, sex), reporter type, patient outcomes, and temporal reporting trends. Categorical variables are presented as frequencies and percentages.

### Integrated system organ class and hierarchical analysis

To thoroughly characterize organ system involvement and clinical manifestations linked to SSRI-opioid and SNRI-opioid combinations, we conducted an integrated analysis across multiple MedDRA hierarchy levels. For each drug combination in both FAERS and JADER, we first performed disproportionality analysis at the System Organ Class (SOC) level using the Reporting Odds Ratio (ROR) and 95% confidence intervals. We then hierarchically mapped the most frequently reported preferred terms (PTs) to their corresponding High-Level Terms (HLTs), High-Level Group Terms (HLGTs), and SOCs. This two-tiered strategy enabled both broad identification of affected organ systems and granular characterization of specific clinical presentations.

### Robust signal detection using multiple algorithms

To improve signal reliability and reduce false positives, we applied a stringent multi-criteria framework requiring concurrent threshold satisfaction across four disproportionality methods: Reporting Odds Ratio (ROR, lower 95% confidence interval >1), Proportional Reporting Ratio (PRR ≥2 with χ^2^ ≥ 4), Empirical Bayes Geometric Mean (EBGM05 > 2), and Information Component (IC025 > 0) (Caster et al., 2014). This conservative approach ensured that only associations consistently identified across complementary statistical techniques were considered validated signals.

### Gender-stratified analysis

Sex-specific subgroup analyses were conducted using FAERS data to evaluate potential differences in adverse event reporting. Disproportionality analysis (ROR with 95% CI) was performed separately for male and female patients exposed to SSRI-opioid or SNRI-opioid combinations. Between-group differences in reporting proportions were assessed using Fisher’s exact test or Chi-square test, as appropriate, with statistical significance defined as p < 0.05.

### Time-to-event analysis

The temporal profile of adverse events following initiation of SSRI-opioid or SNRI-opioid therapy was analysed using FAERS data. Time-to-onset was defined as the interval from therapy start date to adverse event occurrence and categorized into eight periods: 0–30, 31–60, 61–90, 91–120, 121–150, 151–180, 181–360, and >300 days. The distribution of cases across these intervals was summarized using descriptive statistics. All statistical analyses were performed using R software (version 4.3.0).

## Results

### Identification of drugs most frequently associated with serotonin syndrome

**FIGURE 2:**
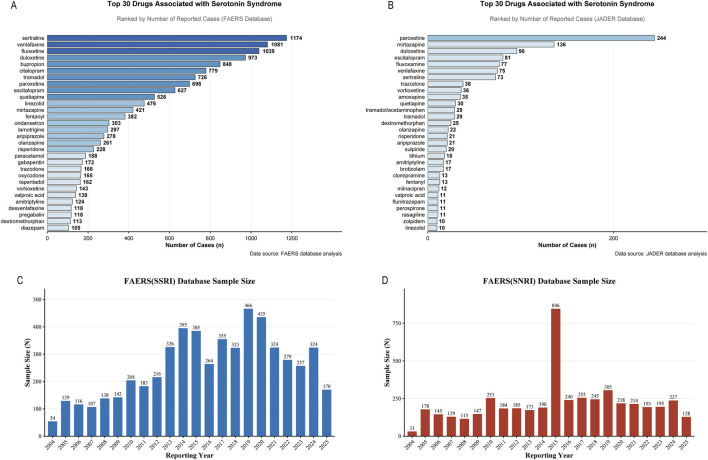
Drugs most frequently associated with serotonin syndrome reports. **(A)** FAERS database (2004–2025). **(B)** JADER database (2004–2025). Bars represent reporting frequencies for individual drug substances. SSRIs, SNRIs and multiple opioid analgesics consistently ranked among the top associated medications in both databases. **(C)** Temporal trends in SSRI-opioid concomitant use reports in FAERS. **(D)** Temporal trends in SNRI-opioid concomitant use reports in FAERS.

Analysis of FAERS and JADER data consistently identified three therapeutic classes most frequently associated with serotonin syndrome: selective serotonin reuptake inhibitors, serotonin and norepinephrine reuptake inhibitors, and opioid analgesics (Figures 2A,B; Supplementary Table S1). In FAERS, sertraline, venlafaxine, fluoxetine, and duloxetine were each linked to over 900 reports of serotonin syndrome, substantially exceeding most other agents. A similar pattern emerged in JADER, with paroxetine, mirtazapine, and duloxetine among the most frequently reported. Of particular perioperative relevance was the consistent presence of multiple opioid analgesics—including tramadol, fentanyl, and oxycodone—among the top substances associated with serotonin syndrome in both databases, suggesting that perioperative opioid exposure may constitute a significant risk factor, especially in patients concurrently receiving serotonergic antidepressants.

### Reporting trends and clinical profiles of concomitant opioid and antidepressant use

Analysis of FAERS data from 2004 to 2025 revealed a substantial overall increase in reports of concomitant opioid and serotonergic antidepressant use. SSRI-opioid combinations rose from 54 cases in 2004 to a peak of 466 in 2019 (Figure 2C), whereas SNRI-opioid reports remained relatively stable until a pronounced surge to 846 cases in 2015, thereafter declining but stabilizing above pre-2014 levels (Figure 2D). Together, these trends indicate a significant increase in reporting of these interactions over the past two decades.

Demographically, most reports involved female patients, with the 18–64-year age group most frequently affected (Table 1). The majority of reports originated in the United States, and physicians were the most common reporter type, reflecting clinical recognition of this interaction. Serious outcomes were frequently reported: hospitalization occurred in 28.2% of SSRI-opioid and 23.0% of SNRI-opioid cases, and death was reported in 22.1% and 11.9%, respectively. These findings underscore the potential severity of this interaction and its implications for perioperative risk management.

**TABLE 1:** Characteristics of concomitant use of antidepressants and opioid analgesics from the FAERS and JADER databases.

Characteristics, number (%)	FAERS-SSRI	FAERS-SNRI	JADER-SSRI	JADER-SNRI
Number of events	5,592	4,806	39	92
Gender				
Male	1810 (32.4%)	1,348 (28.0%)	9 (23.1%)	48 (52.2%)
Female	3,225 (57.7%)	3,172 (66.0%)	30 (76.9%)	42 (45.7%)
Unknow	557 (10.0%)	286 (6.0%)	0	1 (1.1%)
Age(years)				
<2	47 (0.8%)	12 (0.2%)	<10,1 (2.6%)	​
2–11	17 (0.3%)	1 (0.0%)		
12–17	84 (1.5%)	28 (0.6%)	​	​
18–64	2,990 (53.5%)	2,592 (53.9%)	20–69,25 (64.1%)	20–69,43 (46.7%)
65–85	989 (17.7%)	875 (18.2%)	>70,12 (30.8%)	>70,43 (46.7%)
>85	270 (4.8%)	102 (2.1%)		
Unknow	1,195 (21.4%)	1,196 (24.9%)	1 (2.6%)	6 (6.5%)
Weight(Kg)				
<50 kg	154 (2.8%)	80 (1.7%)	5 (12.8%)	13 (14.1%)
50–100 kg	932 (16.7%)	1,126 (23.4%)	3 (7.7%)	37 (40.2%)
>100 kg	157 (2.8%)	229 (4.8%)	31 (79.5%)	​
Missing	4,349 (77.8%)	3,371 (70.1%)	​	42 (45.7%)
Reported countries(Top3)				
United States of America	2,157 (38.6%)	2,808 (58.4%)	Japan (100%)	Japan (100%)
United Kingdom	830 (14.8%)	188 (3.9%)		
France, French Republic	686 (12.3%)	507 (10.5%)		
Reporter				
Consumer	1,067 (19.1%)	1974 (41.1%)	3 (7.7%)	2 (2.2%)
Health professional	439 (7.9%)	335 (7.0%)	2 (5.1%)	12 (13.1%)
Lawyer	100 (1.8%)	23 (0.5%)	NA	NA
Physician	1753 (31.3%)	1,456 (30.3%)	23 (59.0%)	56 (60.9%)
Other	1,190 (21.3%)	484 (10.1%)	NA	NA
Pharmacist	668 (11.9%)	331 (6.9%)	8 (20.5%)	17 (18.5%)
Missing	375 (6.7%)	201 (4.2%)	1 (2.6%)	3 (3.3%)
Outcome				
Congenital anomaly	140 (1.8%)	23 (0.4%)	NA	NA
Death	1762 (22.1%)	718 (11.9%)	7 (11.5%)	16 (9.1%)
Disability	186 (2.3%)	142 (2.3%)	NA	NA
Hospitalization	2,247 (28.2%)	1,390 (23.0%)	NA	NA
Life-threatening	624 (7.8%)	261 (4.3%)	NA	NA
Missing	323 (4.1%)	1,277 (21.1%)	NA	NA
Other serious	2,638 (33.1%)	2,223 (36.8%)	NA	NA
Required intervention	43 (0.5%)	9 (0.1%)	NA	NA

### Adverse event spectrum and system organ class involvement

**FIGURE 3:**
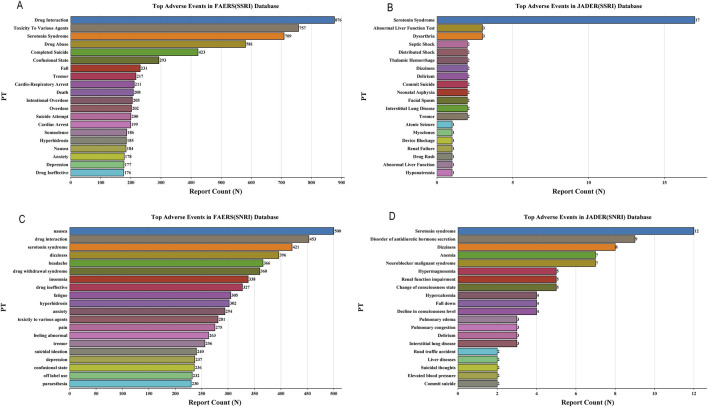
Most frequently reported adverse events for concomitant therapy. **(A)** SSRI-opioid combinations in FAERS. **(B)** SSRI-opioid combinations in JADER. **(C)** SNRI-opioid combinations in FAERS. **(D)** SNRI-opioid combinations in JADER. Data represent the top 20 preferred terms by reporting frequency. SSRI-opioid combinations showed higher proportions of serious outcomes including completed suicide and cardiorespiratory arrest.

Analysis of the top 20 reported adverse events revealed distinct clinical profiles for each drug combination in FAERS (Figures 3A,C; Supplementary Tables S1–S2). For SSRI-opioid concomitant use, frequently reported events included serious and systemic conditions such as Drug Interaction, Toxicity to Various Agents, and Serotonin Syndrome, alongside severe outcomes like Completed Suicide and Cardio-Respiratory Arrest. In SNRI-opioid combinations, Nausea was the most common event, followed by Dizziness, Headache, and Drug Withdrawal Syndrome. JADER data corroborated Serotonin Syndrome as a key event for both classes (Figures 3B,D; Supplementary Tables S3–S4).

**FIGURE 4:**
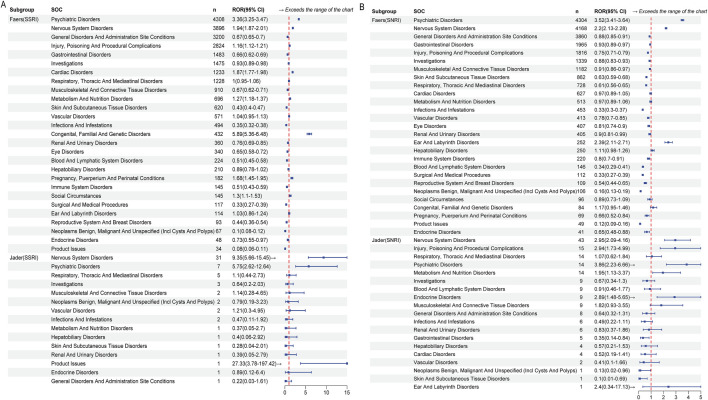
System Organ Class level disproportionality analysis of adverse events associated with SSRI-opioid and SNRI-opioid combinations. **(A)** SSRI-opioid combinations in FAERS and JADER databases. **(B)** SNRI-opioid combinations in FAERS and JADER databases. Forest plots show Reporting Odds Ratios (RORs) with 95% confidence intervals. Both combinations demonstrated strong associations with Psychiatric disorders and Nervous system disorders. SSRI-opioid combinations showed unique signals for Cardiac disorders, while SNRI-opioid combinations exhibited specific associations with Ear and labyrinth disorders. The analysis included reports from 2004 to 2025.

Disproportionality analysis at the System Organ Class (SOC) level revealed both shared and distinct safety signals for SSRI-opioid and SNRI-opioid combinations (Figure 4; Supplementary Table S5). Both drug classes showed strongly significant associations with Psychiatric Disorders (SSRI: ROR = 3.36, 95% CI 3.25–3.47; SNRI: ROR = 3.52, 95% CI 3.41–3.64) and Nervous System Disorders (SSRI: ROR = 1.94, 95% CI 1.87–2.01; SNRI: ROR = 2.20, 95% CI 2.13–2.28), confirming a core neuropsychiatric risk profile. Notable differences included a significant signal for Cardiac Disorders specifically with SSRI-opioid co-administration (ROR = 1.87, 95% CI 1.77–1.98), which was absent for SNRI-opioid use (ROR = 0.97, 95% CI 0.89–1.05). Conversely, SNRI-opioid use was uniquely associated with a signal for Ear and Labyrinth Disorders (ROR = 2.39, 95% CI 2.11–2.71), not observed with SSRIs.

JADER analysis provided external validation, corroborating the strong neuropsychiatric signals and identifying additional significant associations for Endocrine Disorders (ROR = 2.89, 95% CI 1.48–5.65) and Metabolism and Nutrition Disorders (ROR = 1.95, 95% CI 1.13–3.37) specific to SNRI-opioid combinations.

### Hierarchical structure of frequently reported adverse events

**FIGURE 5:**
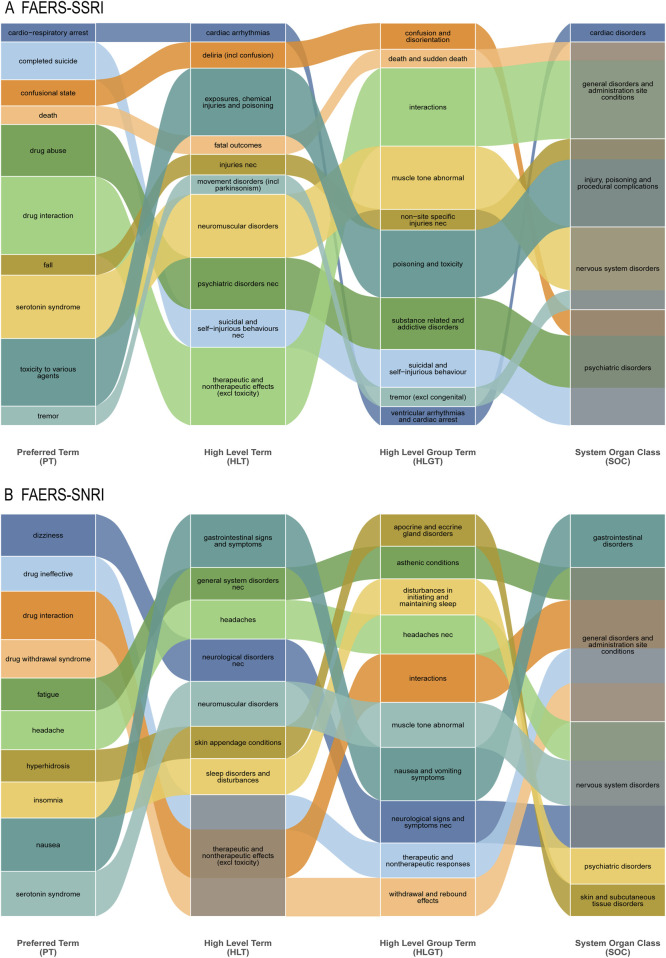
Hierarchical structure of frequently reported adverse events. **(A)** SSRI-opioid combinations. **(B)** SNRI-opioid combinations. Preferred terms (PTs) are mapped to corresponding High-Level Terms (HLTs), High-Level Group Terms (HLGTs), and System Organ Classes (SOCs). The cardiac signal for SSRI-opioid combinations was driven by terms mapping to “Ventricular arrhythmias and cardiac arrest.”

The SOC-level signal profile was strongly supported by specific adverse events at the PT level and their hierarchical groupings. For SSRI-opioid combinations, the cardiac disorder signal was reflected in PTs such as Cardio-Respiratory Arrest and Death, which map to the HLT “Ventricular arrhythmias and cardiac arrest” (Figure 5A). Similarly, prominent neuropsychiatric signals were underpinned by high-frequency PTs including Serotonin Syndrome (HLT: Neuromuscular disorders), Completed Suicide, and Confusional State.

For SNRI-opioid combinations, the unique SOC signal for Ear and Labyrinth Disorders aligned with the high frequency of Dizziness—a common vestibular symptom—which was the most frequently reported nervous system event (Figure 5B). Furthermore, JADER-identified signals for Endocrine and Metabolism disorders were contextually supported in FAERS by PTs such as Drug Withdrawal Syndrome, a condition often associated with autonomic and metabolic dysregulation.

In summary, SOC analysis not only confirms shared serious neuropsychiatric risks but also highlights distinct organ system involvement—cardiac with SSRIs and otological/endocrine-metabolic with SNRIs—offering a more nuanced understanding of the safety profiles of these concomitant therapies.

### Robust signal detection using multiple algorithms

**FIGURE 6:**
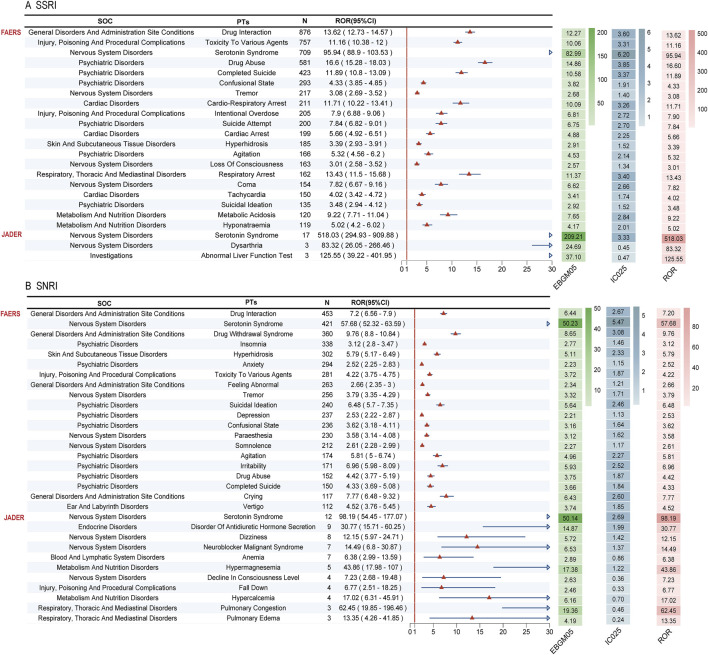
Multi-algorithm signal detection for serotonin syndrome and related adverse events. **(A)** SSRI-opioid combinations in FAERS and JADER databases. **(B)** SNRI-opioid combinations in FAERS and JADER databases. Serotonin syndrome demonstrated exceptionally high reporting odds across all statistical methods and both databases, with ROR values exceeding 50 for all combinations. Additional significant signals included drug interaction, cardiorespiratory arrest, and drug withdrawal syndrome.

Application of a multi-algorithm disproportionality framework (ROR, PRR, EBGM, IC) consistently identified significant safety signals for both SSRI-opioid and SNRI-opioid combinations across FAERS and JADER. The most pronounced signal across all analyses was for Serotonin Syndrome, which showed exceptionally high reporting odds in both databases (FAERS-SSRI: ROR = 95.94, 95% CI 88.9–103.53; FAERS-SNRI: ROR = 57.68, 95% CI 52.32–63.59; JADER-SSRI: ROR = 518.03, 95% CI 294.93–909.88; JADER-SNRI: ROR = 98.19, 95% CI 54.45–177.07) (Figure 6).

Beyond this expected pharmacodynamic interaction, robust signals were detected for Drug Interaction for both drug classes. SSRI-opioid use was strongly associated with serious and fatal outcomes, including Cardio-Respiratory Arrest (ROR = 11.71, 95% CI 10.22–13.41), Respiratory Arrest (ROR = 13.43, 95% CI 11.5–15.68), and Completed Suicide (ROR = 11.89, 95% CI 10.8–13.09). In contrast, a defining signal for SNRI-opioid combinations was Drug Withdrawal Syndrome (ROR = 9.76, 95% CI 8.8–10.84). JADER data further validated SNRI-specific signals, including Disorder of Antidiuretic Hormone Secretion (ROR = 30.77, 95% CI 15.71–60.25) and electrolyte imbalances such as Hypermagnesemia (ROR = 43.86, 95% CI 17.98–107).

The consistency of these signals across four distinct quantitative methods confirms that concomitant use of serotonergic antidepressants and opioids is associated with a broad spectrum of severe adverse events, encompassing serotonergic, cardiac, respiratory, metabolic, and withdrawal-related complications.

### Gender-stratified and time-to-onset analyses

**FIGURE 7:**
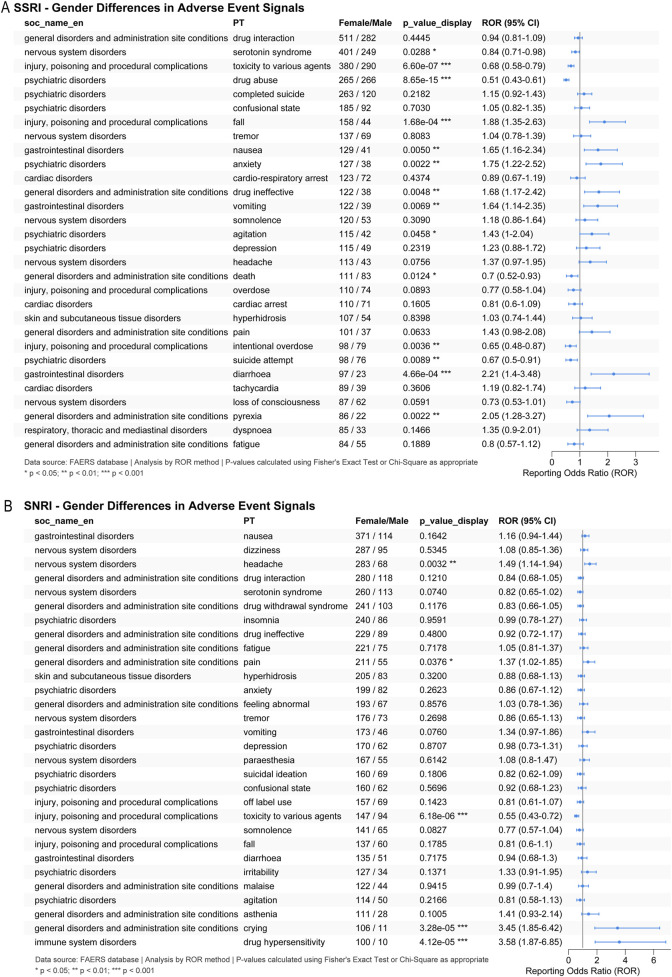
Gender-stratified analysis of serotonin syndrome reporting. **(A)** SSRI-opioid combinations. **(B)** SNRI-opioid combinations. Reporting Odds Ratios (RORs) with 95% confidence intervals are shown separately for male and female patients. For SSRI-opioid combinations, males showed a statistically significant higher reporting proportion for serotonin syndrome (ROR 0.84, 95% CI 0.71–0.98; P = 0.0288).

Gender-stratified disproportionality analysis revealed a distinct pattern for serotonin syndrome reporting between SSRI-opioid and SNRI-opioid combinations. For SSRI-opioid combinations, the ROR for serotonin syndrome was 0.84 (95% CI 0.71–0.98; p = 0.0288) for females versus males, indicating a statistically significant lower reporting proportion among females despite a higher absolute number of cases (Figure 7A). This suggests that male patients may be at relatively higher risk for serotonin syndrome with SSRI-opioid co-administration after adjusting for overall reporting patterns. In contrast, no statistically significant gender difference was observed for SNRI-opioid combinations (ROR = 0.82, 95% CI 0.65–1.02; p = 0.0740; Figure 7B), though the point estimate similarly suggested lower female reporting.

**FIGURE 8:**
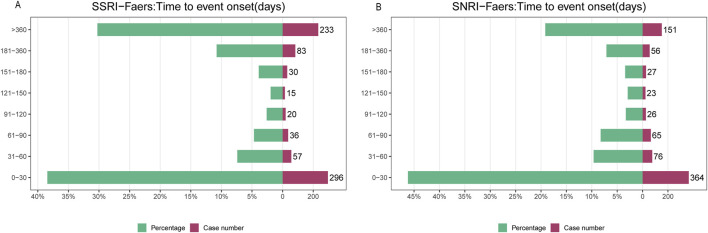
Time-to-onset analysis of adverse events Cumulative incidence curves for **(A)** SSRI-opioid and **(B)** SNRI-opioid combinations. While most events occurred within the first 30 days of therapy, substantial proportions emerged after extended treatment durations (>300 days: 30.26% for SSRI-opioid, 19.16% for SNRI-opioid combinations).

Time-to-onset analysis of all reported adverse events revealed consistent patterns across both drug classes (Figure 8; Supplementary Table S7). Most events occurred early in treatment, with 38.44% of SSRI-opioid and 46.19% of SNRI-opioid events reported within the first 30 days. However, a substantial proportion of cases (30.26% for SSRI-opioid and 19.16% for SNRI-opioid combinations) emerged after more than 300 days of therapy, indicating that adverse event risks persist throughout the treatment course.

## Discussion

This multinational pharmacovigilance study establishes a significant association between concomitant opioid-serotonergic antidepressant use and serotonin syndrome risk, with distinct safety profiles emerging between SSRI- and SNRI-opioid combinations. These findings carry immediate relevance for perioperative medicine, where opioid administration remains fundamental to analgesia, yet systematic evidence regarding interaction risks with commonly prescribed antidepressants has been limited.

The strikingly high disproportionality signals for serotonin syndrome across both FAERS and JADER databases—with ROR values among the highest observed—strongly support a clinically meaningful interaction. While serotonin syndrome is a recognised pharmacological entity, its prominence in a perioperative context underscores an underappreciated risk. Mechanistically, this interaction is biologically plausible: multiple opioids frequently employed during anaesthesia, including tramadol, fentanyl, and oxycodone, exhibit serotonergic activity through reuptake inhibition or enhanced release. When combined with SSRIs or SNRIs, these agents may produce additive or synergistic increases in synaptic serotonin, particularly under the physiological stress of surgery, where autonomic regulation may be compromised (Caster et al., 2014; Smischney et al., 2018; Warner et al., 2017; Evans, 2007).

Beyond serotonin syndrome, our analysis delineates broader and divergent safety profiles. SSRI-opioid combinations demonstrated significant associations with serious cardiac events, including cardio-respiratory arrest and tachycardia, suggesting these combinations may pose specific risks to cardiovascular stability in vulnerable patients (Pillinger et al., 2025; Mikkelsen et al., 2023; Chen et al., 2023; Edinoff et al., 2021). In contrast, SNRI-opioid use was characterised by prominent signals for drug withdrawal syndrome and autonomic manifestations such as hyperhidrosis, possibly reflecting the additional noradrenergic activity of SNRIs (Beyer et al., 2017). These differential patterns emphasise that the risks of combined therapy extend beyond serotonin syndrome alone and may inform agent selection in patients with specific comorbidities.

The observed gender difference in serotonin syndrome reporting with SSRI-opioid therapy—showing a higher reporting proportion in males despite greater absolute numbers in females—suggests potential sex-based differences in serotonergic vulnerability. This finding warrants further exploration into possible pharmacogenetic, metabolic, or hormonal influences that may modulate risk (Young et al., 2009; Kirchheiner et al., 2005; Ekhart et al., 2018; Herzog et al., 2019).

Temporal analysis further contextualises these risks. Although a substantial proportion of adverse events occurred within the first 30 days of combined therapy, a significant number emerged after extended treatment durations, including beyond 300 days. However, this finding of “delayed onset” requires cautious interpretation. While it suggests that vigilance is required throughout the treatment course, these late-reported events likely do not represent a slow accumulation of toxicity. Instead, they may reflect: (1) reporting lags (a discrepancy between the actual event date and the report date); (2) the late introduction of a new precipitating factor, such as dose escalation or the addition of a third serotonergic agent during chronic maintenance therapy; or (3) physiological changes in the patient (e.g., deteriorating renal function) that altered drug pharmacokinetics over time. Therefore, clinicians should remain alert to serotonin syndrome not only during induction but also whenever therapeutic regimens are modified in chronic users (Wei et al., 2022; Hawkins et al., 2013; Herzig et al., 2022).

When interpreting these findings, several methodological considerations should be noted. The use of multinational databases and a multi-algorithm disproportionality framework enhances the robustness and generalisability of signal detection. However, as with all pharmacovigilance studies, potential biases including under-reporting, confounding by indication, and variable data quality remain inherent limitations (Ma et al., 2025; Hauben and Zhou, 2003; Coste et al., 2023). The absence of detailed clinical data, such as opioid dosing, specific anaesthetic techniques, and genetic profiles, precludes causal inference and precise risk stratification. Nevertheless, the consistency of signals across complementary databases and analytical methods supports the validity of the primary conclusions.

### Clinical implications

The findings from this multinational pharmacovigilance study highlight several critical imperatives for enhancing patient safety. First, systematic risk identification and preoperative vigilance must be prioritized. Integrating formal medication reconciliation into standard preoperative checklists to explicitly screen for concomitant serotonergic antidepressant and opioid use is essential. Identifying such combinations should trigger flagging systems and heightened awareness among clinicians, given their strong association not only with serotonin syndrome but also with severe cardiac and autonomic adverse events, necessitating careful monitoring throughout the perioperative journey.

Second, tailored monitoring and analgesic selection strategies should be guided by the distinct risk profiles observed. For patients on SSRI-opioid combinations, enhanced and prolonged cardiovascular monitoring is warranted due to signals of cardiorespiratory arrest and tachycardia. In contrast, those on SNRI-opioid therapy require vigilance for autonomic manifestations and withdrawal symptoms, alongside proactive patient counselling. Furthermore, when analgesia is required in these high-risk patients, prescribers should exercise caution with serotonergic opioids (e.g., tramadol, fentanyl) and prioritize non-serotonergic alternatives (e.g., morphine) or multimodal non-opioid regimens to mitigate interaction risks.

Finally, sustained vigilance and institutional protocol development are paramount. The substantial proportion of adverse events occurring after prolonged therapy underscores that risk persists well beyond treatment initiation, demanding a high index of suspicion during long-term follow-up. To standardize care, hospitals should develop evidence-based guidelines encompassing the entire patient pathway—from preoperative screening and intraoperative management to post-discharge monitoring and the acute management of serotonin syndrome. Implementing such a structured, system-wide approach is crucial to mitigating the risks of this common yet potentially severe drug interaction.

### Limitations

Several limitations inherent to pharmacovigilance studies must be acknowledged when interpreting our findings. First, and most fundamentally, spontaneous reporting systems like FAERS and JADER are subject to under-reporting (the Weber effect), reporting bias, and incomplete clinical information. As these databases lack a denominator (the total number of patients exposed), the Reporting Odds Ratio (ROR) represents a measure of statistical association (signal strength) rather than a definitive biological causality or a precise incidence rate.

Second, our case selection strategy prioritized specificity over sensitivity. By restricting our analysis to reports where both opioids and antidepressants were listed as “Primary Suspect” or “Interacting,” we aimed to minimize false-positive associations. However, this conservative approach inevitably excluded cases where these drugs were recorded as “Concomitant,” potentially underestimating the true burden of the interaction and biasing our results towards more severe or clinically obvious cases recognized by reporters.

Third, the influence of confounding factors cannot be fully eliminated. Confounding by indication (e.g., patients with severe pain and depression may be inherently sicker) and polypharmacy are common in this population. While the high magnitude of the detected signals (ROR >50) suggests a true drug-drug interaction effect, the inability to statistically adjust for disease severity, comorbidities, and other concurrent medications remains a limitation of the open-access pharmacovigilance data structure.

Finally, regarding database selection, while FAERS and JADER offer valuable insights into US and Japanese populations, the exclusion of other large international databases, such as the WHO VigiBase (due to access restrictions), may limit the global generalizability of our findings to other regions with different prescribing behaviors. Future studies incorporating broader datasets and structured clinical records are warranted to validate these signals.

## Conclusion

This large-scale pharmacovigilance analysis demonstrates that concomitant use of opioid analgesics and serotonergic antidepressants is associated with a significant risk of serotonin syndrome and other serious adverse events. The safety profiles of SSRI-opioid and SNRI-opioid combinations are distinct, with SSRI combinations showing stronger cardiac signals and SNRI combinations exhibiting more prominent withdrawal and autonomic manifestations. Gender-based differences in serotonin syndrome reporting and the persistence of risk throughout the treatment course further characterise these interactions. These findings underscore the importance of vigilance and individualised risk assessment in patients receiving these medication combinations, particularly in the perioperative setting.

## Data Availability

The original contributions presented in the study are included in the article/Supplementary Material, further inquiries can be directed to the corresponding authors.
